# Danish translation of a physical function item bank from the Patient-Reported Outcome Measurement Information System (PROMIS)

**DOI:** 10.1186/s40814-017-0146-7

**Published:** 2017-05-31

**Authors:** Christina W. Schnohr, Charlotte L. Rasmussen, Henning Langberg, Jakob B. Bjørner

**Affiliations:** 1 Section of Social Medicine, Department of Public Health, University of Copenhagen, Oester Farimagsgade 5, 1014 K Copenhagen, Denmark; 2 Optum Outcomes, 24 Albion Road, Lincoln, RI 02865 USA

**Keywords:** Patient reported outcomes, PROs, PROM, PROMIS, Cognitive testing, Translation methodology

## Abstract

**Background:**

The Patient-Reported Outcome Measurement Information System (PROMIS) is an assessment system that aims to provide more valid, reliable, responsive, and precise patient-reported outcome (PRO) measures than has been previously available. This paper documents the translation of the Physical Function item bank into Danish.

**Methods:**

We followed the PROMIS standard procedure, including: 1) two independent translations, 2) back translation, 3) independent reviews of translation quality, and 4) cognitive interviews with a representative sample of the adult population from the municipality of Copenhagen. After each phase, the new information was reviewed and the Danish version of the PROMIS Physical Function items was revised, if warranted.

**Results:**

Relatively few problems were related to translation in itself and such problems could be fixed by changes in item wordings to fit the Danish context. Cognitive testing revealed problem of a general issue: annoyance in case of mismatch between respondents’ functional level and question difficulty, problems imagining performance on activities that the respondents did not usually do, and uncertainty whether mobility aids (e.g., canes and walkers) should be considered when performing an activity. Solutions to the more general issues would require revisions to the original items.

**Conclusions:**

The standard translation methodology was successful in eliminating problems in translation, and pointed to problems of a general issue in some of the original questions, producing translated Danish versions of the PROMIS Physical Functioning items. Translation and validation studies provide a valuable source when revising and improving PROs in a clinical setting or for research. The present paper exemplifies this with experiences from Denmark. The study describes how the use of PROs when measuring physical functioning in a Danish context can be improved—hence improving the items used for research, future trials and in clinical settings.

## Background

Over the past 20 years, Patient Reported Outcomes (PROs) have gained increasing importance as measures of health, wellbeing, and function in medical and social sciences. PROs are important supplements to traditional clinical measurements, by allowing the patient report to be the primary source used to assess the person’s health and to assist patient-doctor communication [1].

Physical function (PF) is one of the most common PROs used for evaluation of health outcomes and for health risk assessment, which makes PF one of the most important PRO domains. Even though there are already a number of validated and well-used instruments on PF, most instruments cover a narrow span of PF, primarily focusing on severe physical dysfunction. Thus, general population samples will often include a high percentage of persons achieving best possible score on traditional PF measure (ceiling effect). For many instruments, noticeable ceiling effect is even seen in patient samples because the items included are not challenging enough for the part of the patient samples with the best PF. Ceiling (and floor) effect diminish the usefulness of a measure. In particular, ceiling effect limits the ability of a tool to detect improvement over time (responsiveness). In order to meet these methodological challenges, the National Institutes of Health (NIH) launched an initiative to develop a measurement system that is more valid, reliable, and responsive: The Patient-Reported Outcomes Measurement Information System (PROMIS®) [2, 3].

The PROMIS project developed a set of item banks for health domains such as: PF, fatigue, pain, and depression [2, 3]. The project originally developed item banks within 26 health domains. Each item bank contains a set of items designed to measure the same latent construct, which is called a “domain” (e.g., PF). The constructs were chosen from the conceptual framework on health advocated for by the World Health Organization (WHO) with three overall domains; physical, mental, and social health. Those domains were thought to represent general constructs for measuring health and wellbeing. The item banks were aimed to be generic, that is, relevant for all respondents regardless of diagnoses, age, gender, ethnicity, etc. [4]. The item banks included between 6 and 121 items in total. Each item bank was carefully developed and tested using cognitive debriefing and a thorough psychometric analysis that included classical psychometrics, factor analysis, and item response theory (IRT) modeling [5]. In applied research, the item banks permit different modes of administration: as a fixed standard short form, as a targeted short form selected by the researcher, or as a Computer Adaptive Test (CAT) [5]. Short forms in this context are questionnaires of 6 to 8 or 10 fixed items from the item bank. CAT is a form of computer-based testing that adapts to the respondent’s ability level, which is also called tailored testing. After each question, the person’s health score is re-estimated, and the next item is selected to provide maximum possible information at this particular health level. Testing typically stops when the score is estimated with sufficient precision, which usually happens after responding to 5–7 questions. Thus, the CAT version also decreases the response burden for patients [6] in comparison to other instruments of 12 items and more.

The PF item bank was one of the first item banks to be developed [7, 8]. It has been linked to established measures such as the Health Assessment Questionnaire (HAQ) [9] and the SF-36 physical function scale [10], allowing researchers to directly compare results to results from these tools. Although the PROMIS PF item bank includes the same domains as the HAQ and the Short Form 36 (SF-36) Physical Function scales, it covers a much broader score range by including both “very easy” (e.g., “are you able to turn from side to side in bed”) and “difficult” items (“are you able to run 10 miles”).

Earlier studies have described findings from the translation of PROMIS item banks. These studies have shown the benefits from including both quantitative and qualitative assessments [11, 12]. Studies describing the results and experiences with the translation procedures are an important part of the international comparability of the PROMIS measurement system.

The purpose of the present study was to document the translation of the 121 PROMIS items on PF into Danish. The present paper describes the qualitative aspects of the methodology to attain cross-language equivalence: 1) semantic/linguistic (making sure that the meaning of the item is the same in the source and the target language), 2) content (making sure that the item is relevant in both cultures), and 3) conceptual (making sure that the item measures the same theoretical construct in the target as well as the source item).

## Methods

### Translation procedure

**Fig. 1:**
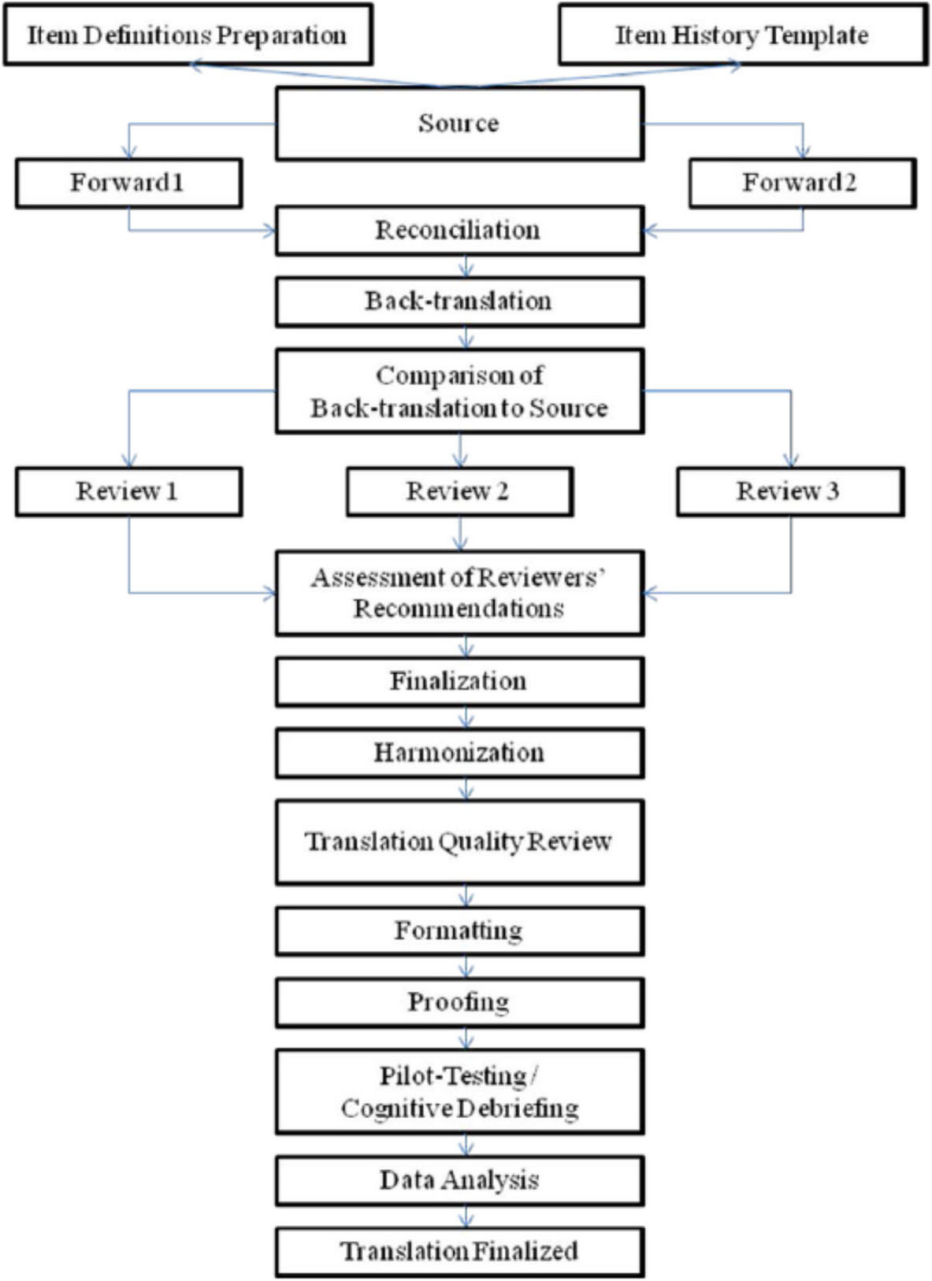
FACIT translation methodology chart

Translation of the PROMIS PF item bank used standard multilingual translation methodology [11, 13, 14] including several forward and backwards translations, independent assessment of translation quality, and pilot testing including cognitive debriefing (see Fig. 1):

1. Two parallel forward translations were conducted from English to Danish. The translators were native speaking Danes with university degrees in English.
2. The translation team (CWS, HL, and JBB) reconciled the two forward translations, evaluated all discrepancies and created a hybrid version.
3. Back-translation was done by a bilingual American residing in Denmark, who did not have access to the original version.
4. The translation project manager (CWS) compared source items and the back-translated English version, and identified discrepancies. In case of discrepancies, the two forward translations and the hybrid version were analyzed to evaluate whether the problem concerned the forward or the back translation.
5. Three independent native Danish speaking experts (one linguist, one expert in development of questionnaires, and one expert in PF) examined all of the preceding steps, and made recommendations on finalized items.
6. The final version of the items were decided by the translation project manager (CWS) and the language coordinator (JBB) who were both native Danish speakers who had previously lived and worked in the UK for two years and the US for 4 years respectively. For all items, the rationale for the choices made was documented. Particularly careful documentation was made if decisions deviated from the recommendations proposed by the expert review.
7. The translation project manager performed a quality assurance before sending a completed suggestion to the PROMIS’ translation center. Here, the PROMIS Statistical Center did a quality review comparing the original items and the back-translated items evaluating comments provided.
8. A cognitive testing and linguistic validation was then performed (see detailed description below).
9. The results from the cognitive testing were used to finalize the questions in a Danish cultural and language context.
10. A finalized Danish translation was submitted to (and approved by) the PROMIS Statistical Center.

### Pilot testing and cognitive debriefing

We aimed to include approximately 30 respondents. The respondents were recruited from rehabilitation centers in the municipality of Copenhagen. Initial recruitment was performed by the staff of the rehabilitation centers. All participants were informed that participation was voluntary and that refusal to participate would have no impact on their treatment. If respondents agreed to participate, interviews were performed at the rehabilitation center by CWS with an interview time frame of 45–55 min. To avoid excessive response burden, we developed five blocks of different items so that the interview for each participant concerned 20% of the total number of items in the item bank. The number of participants was recruited to ensure that each question was evaluated by at least five participants. In case of question revisions, new participants were recruited to ensure that the revised questions were also evaluated. When evaluating the need for further recruitment, we assessed whether the last interview for the particular item block had achieved new information. If not, recruitment was stopped.

After completing 22 interviews, adjustments were made to the response categories and on some items. Thereafter, another eight interviews were completed to test the modified versions of the items and responses. Final minor revisions were done for three items, so brief cognitive interviews were performed with five new respondents before revising and submitting a finalized Danish version to the PROMIS Statistical Center. Content exhaustion was demonstrated since the last five interviews resulted in no new information.

The interviews focused on the respondent’s interpretation of each question and their response strategies. Right after answering each item, the respondent was probed with respect to four issues [15]:

- How the respondent understood the item,
- What strategies were used to collect information from memory,
- What kind of evaluation took place to determine whether the memorized information was relevant and sufficient, and,
- How the final response was chosen.

After a short general introduction of the project, the cognitive interview consisted of all respondents completing a version of the questionnaire containing 20–25 of the items in the item bank. The filled-in questionnaire then worked as an interview guide of the cognitive debriefing based on the issues a, b, c and d mentioned above. The questionnaires were administered via paper and pen, and the interviews were recorded from the time the respondent had filled in the questionnaire. The interviews were fully transcribed, read and kept in electronic files (the original transcriptions as Word files as well as integrated in NVivo). Interviews were analyzed by CWS and the interpretations of all findings were discussed within the translation team. Data analysis was performed in two rounds: 1) Initial analysis focused on item-specific problems in interpretation and response. These specific findings were used to revise translations. 2) A second round of analysis identified themes of interpretation and reaction to items that were common across several items. Some of the more general and conceptual findings added to the understanding of the functioning of the item banks in a Danish context, while some findings concerned general measurement issues. Both types of findings are presented below.

## Results

The respondents varied across age, cohabitation status and vocational training, but were all patients within a rehabilitation program; elder care and cancer patients. The age range was 40–90 (mean age = 70, standard deviation SD = 12.2, Table 1).

**Table 1:** Demographic information

		*N*
Age	40–49	2
	50–59	3
	60–69	9
	70–79	9
	80–90	6
Gender	Men	10
	Women	20
Cohabitation	Living alone	23
	Living with spouse/partner	7
Vocational training	No education	4
	Short (<2 years)	9
	Craft training	8
	Professional education/bachelor	6
	University: Master/PhD	3
Rehabilitation center	Cancer rehabilitation	13
	Elder care	17

### Item specific findings and solutions

The interviews revealed several item-specific problems of interpretation that could be resolved through revisions of the item text:

- When asked about the item *Are you able to carry a laundry basket up a flight of stairs?* several respondents expressed uncertainty whether the laundry basket was full or empty, as they considered it a big difference in assessing whether they could carry it one flight of stairs. Therefore, the item was rephrased to (Danish translation) *Are you able to carry a basket of laundry up a flight of stairs* making it clear that the basket had laundry in it.
- When asked about the item on taking care of personal needs *Does your health now limit you in taking care of your personal needs (dress, comb hair, toilet, eat, bathe)?* several respondents were confused by the examples given, since the translation of “personal needs” was understood as a question on maintaining personal hygiene, and therefore not well matched to the examples given. The example served more as confusion, by including eating and getting dressed. Therefore, the example “eating” was omitted from the item.
- When asked about the item *Are you able to lift one pound to shoulder level without bending your elbow?* no respondents immediately understood the question, and several had not replied to it. All respondents tried to do the activity, and asked the interviewer whether it was the correct way (the interviewer herself was in doubt about what activity was addressed by the description given). Therefore, the item was changed from “without bending your elbow” to “with a straight arm”, which is a more natural expression in Danish.

### Response categories

One set of response categories for items on limitations in physical activities served as an independent challenge to the translation procedure, as the expert review did not agree with the recommendation from the translation team. Therefore, two sets of responses choices were tested with respondents (Table 2).

**Table 2:** Response categories tested (in Danish, authors translations in parenthesis)

Slet ikke, i mindre grad, i nogen grad, i høj grad, fuldstændig begrænset
(Not at all, To a lesser degree, To some degree, To a high degree, Totally limited)
vs
Slet ikke, Lidt, Noget, Meget, Kan ikke
(Not at all, A little, Some, A lot, Cannot)

One set of response choices repeated the word “limited” to emphasize the link to the stem item. The other set of response choices were short (one to two words) statements. Both versions were easily understood, perceived appropriate for the questions, and relevant to the respondents. Approximately 25% of the respondents favored the shorter version (typically because of its brevity and simplicity), 25% favored the longer version, and approximately 50% were indifferent. Based on the evaluations and the conclusion that both versions were appropriate in relation to the items, the shorter version was chosen. Examples of the items in full length and their response categories are available online [16].

### General issues

We identified three general issues across the item bank:

#### Theme #1: Adapting to the level of the respondent

The interviews clearly showed that respondents found it irrelevant to reply to questions that were either 1) way too difficult or 2) far too easy for them to do. While the respondents did not indicate problems answering such very easy or very difficult questions, slight probing revealed the respondents’ annoyance with questions that were ill matched to their ability level (Table 2).

#### Theme #2: With or without aids?—lack of explicit context

The cognitive debriefing showed that the respondents differed in their interpretation whether the activities could be performed with or without the use of aids such as a cane or a walker (zimmer frame). Some respondents perceived the questions to refer to physical activities as being done with aids; other respondents assumed that the activities should be done without aids, while still other respondents did not reply due to uncertainty (Tables 3 and 4). Some respondents decided for themselves whether the activity was done with or without aids (Tables 3 and 4). Finally, some respondents replied that they were able to do all the listed activities, even though they were clearly disabled. One example was a woman living alone, but was not able to walk without her zimmer frame. She had a variety of mobility aids attached to the zimmer frame, making it possible for her to move around her house and she did not feel constrained when doing daily activities (e.g., having to pick up something from the floor), even though she was unable to do this without aids.

**Table 3:** Three quotes regarding adaptation to the level of the interviewed

*Q: Are you able to stand without losing your balance for several minutes?*
*A: Yes*
*Q: What were you thinking?*
*A: That it is a weird question. If I cannot stand without losing my balance I am either ill or I have had too much wine…*
*Q: Are you able to pull on trousers?*
*A: Yes*
*Q: What were you thinking?*
*A: That it was a silly question, who else would put them on me…*
*Q: Are you able to run or jog for two miles?*
*A: I am not Prince Frederik! (referring to the Danish Crown Prince who is very active and did an Ironman recently when interviewed)*

**Table 4:** Two quotes regarding aids

*Q: Does your health now limit you in going for a short walk (less than 15 minutes)?*	
*A: I have not answered, as I don’t know if you mean using my aids?*	
*Q: What do you think?*	
*A: Well, I stopped at that question, because I don’t know if you mean with or without my stick. If it is with the stick, I can walk quite far, but without my stick, I cannot do it.*	
*Q: Well, if I challenge you and say that you have to tick one of the boxes?*	
*A: I cannot answer that when I don’t know if it is with aids.…*	
*Q: Does your health now limit you in going for a short walk (less than 15 minutes)?*	
*A: Yes*	
*Q: You thought with a stick?*	
*A: Yes. But it does not say so. Should I just write it next to?*	
*Q:Well, it is not a good question, when you have to write something next to it. But it is good to know, that you use a stick, so you are thinking that it is with a stick.*	
*A: Yes*	

#### Theme #3: Able or unable to do—a matter of non-figurative thinking

Another general finding was that responses to some questions depended on the respondents’ ability to imagine performing an activity that they did not usually do, i.e., whether they were able to think non-figuratively. This ability turned out to be important for the response. It became evident, that there were two reasons for respondents to reply that they were “unable” to perform a given physical activity; either that they had not done it (ever or for a long time), or that they knew that they were not able to do it. The latter option was chosen because of known lack of ability, or that respondents knew of similar activities that they were unable to perform and compared.

Table 5 is from respondents able to think non-figuratively. The quotes illustrate how the respondents did not need to be able to jump in one place or take a bath to know whether they are able to do so. In contrast, Table 6 is from respondents not able to think non-figuratively. The quotes illustrate how some respondents lacked the ability to imagine activities that they had not actually performed or were able to compare them to known activities. The last quote in Table 6 was from a middle aged woman. Judging from her physique and her responses to other questions, she should clearly be able to hold a hammer and pound a nail. Her reply was due to her lack of experience with this particular activity, rather than her self-assessment of muscle strength and joint function, and that she was not able to imagine the activity.

**Table 5:** Two quotes regarding non-figurative thinking

*Q: Are you able to jump in place?*	
*A: No*	
*Q: What did you think?*	
*A: Then I will lose my balance*	
*Q: All right*	
*A: Not that I have tried, because I have not dared to try…*	
*Q: Are you able to button your shirt?*	
*A: It is not a problem, I am sleight*	
*Q: What did you think?*	
*A: To button them. I don’t have shirts, but I have pants.*	
*Q: You reply to the question even though you don’t wear shirts?*	
*A: Yes*

**Table 6:** Two quotes regarding non-figurative thinking

*Q: Are you able to water a house plant?*	
*A: I don’t know, because Lisa does that…*	
*Q: Are you able to use a hammer to pound a nail?*	
*A: I have answered unable to do, because I have never done it. I wouldn’t be able to do it at all.*	
*Q: Do you think that you don’t have the motor skills, or have you just never tried it?*	
*A: No, well, I am sure I would miss*	

## Discussion

An important finding of the cognitive testing was that even though the items were developed in an American context, only a few cases pointed to differences in cultural understandings in Denmark. Generally, the Danish versions of the items were easily understood and easy to administer. Only in one case, we found different cultural understanding in a description of an activity. In this case (an item regarding personal care) the Danish translation was not well in alignment with the examples, which led to confusion. Thus, the examples were revised to match the Danish context.

Like in previous translation studies [12], the change of pounds and miles into kilograms and (kilo) meters sometimes resulted in awkward distances. The item concerning ability to run 5 miles was translated into the distance 8 km. However, 100 yards was translated to 100 meters (approximately 110 yards) since the distance 92 m was deemed too odd to include. Similarly, the American door-knob does not exist in Denmark, and “door-knob” was translated to “door handle”. A similar choice was made in the Dutch-Flemish translation [12]. The Dutch translation team found the item “Does your health now limit you in putting a trash bag outside?” irrelevant in a Dutch context, as trash bags are hardly used any more in the Netherlands and Flanders [12]. This is not the case in Denmark, where the item was found relevant.

For several English words, the translation team had to choose between several possible Danish translations that were conceptually equivalent. Thus, the English phrase “Are you able to…” could be translated into Danish phrases similar to “Can you…” (the most common form when speaking) and “Do you have the abilities to…” (the most common form in writing). Since there was no conceptual difference between the Danish phrases, the latter was chosen since we expect the questionnaire to be read (from a screen or paper) rather than administered by interview. In contrast, the Dutch-Flemish translation chose the most commonly used wording in everyday speech [12].

In several cases, the choice of Danish wording lead to interpretational errors or uncertainties that were unexpected by the translators and researchers (e.g., the question whether a laundry basket is full or empty). Once identified by cognitive testing, these uncertainties were relatively easy to resolve, which illustrated the benefits of thorough translation processes in translating item banks.

However, our general findings point to issues that are harder to reform: the breadth of item difficulty, uncertainty about the use of aids, and the inclusion of items that pertain to activities the respondent is unfamiliar or may reply that they are unable to do for reasons unrelated to physical functioning. We will discuss the implication of each of these results.

The PROMIS item bank has successfully included items concerning a broad range of activity levels. Thus, most respondent may encounter items about activities that are either much too difficult or much too easy for them. While respondents generally understand these items, having to answer items that are felt to be irrelevant can be annoying and lead to less care in responding. These results underline the importance of items being administered as a CAT, which quickly adapts to the respondent’s level of physical functioning by nature and administers items about activities of appropriate difficulty [6, 8]. Current quantitative validation is taking place while collecting data to test for differential item functioning (DIF) and to test the appropriateness of the US IRT calibrations, before CAT can be implemented in a Danish context. However, currently the short forms can be used and are available from the PROMIS website.

The second general finding concerned the uncertainty whether the ability of performing the different physical activities were to be assessed with or without aids. The issue whether an activity could be performed with the use of aids was relevant to the elderly population of our study. For other samples, the issue may be less relevant. Some physical functioning questionnaires directed at elderly populations have made the use of aids explicit and offers scoring options to use this information [9]. Other PF scales do not adapt this approach [17]. Our results suggest that this issue may be a source of systematic or random error when PROMIS is distributed to patients using aids for their everyday physical functioning, such as geriatric patients. We recommend that this issue is further explored in quantitative studies of elderly populations to see if the potential uncertainty induces bias or impacts reliability.

Finally, the cognitive interviews suggested that respondents were not always able to answer questions about their ability to perform activities that they had never tried to do. If respondents had never tried to do the exemplified activities, some responded that they were unable regardless of their level of ability. Others were able to imagine the demands proposed, and responded to the questions about activities that were hypothetical for them. In any given situation, the ability to respond to questions on hypothetical activities also depends on the cognitive level of the respondents. The impact of non-figurative thinking may be expected to particularly concern the interpretation of the responses “unable to do”; as this response can be chosen either because 1) the person IS in fact unable to do the activity or 2) that the respondent is not able to imagine the activity and therefore replies “unable”. It can be hypothesized that there is an over-reporting of the biased response in lower educated respondents. We recommend that the potential impact of non-figurative thinking on responses to PF items is evaluated in future quantitative studies.

One limitation of the present study was that the respondents were relatively older in comparison to the background population, as well as being primarily from an urban population. Furthermore, there was a narrow variation in geographical location of the respondents, since most (two thirds) stemmed from the capital. Other than this homogeneity, the respondents were similar to the general population with respects to socio-economic status, gender, and marital status.

## Conclusions

The present study made use of the standard translation methodology for PROMIS studies, which was successful in eliminating problems both in the linguistic content and conceptual translation, and pointed to general types of problems in some of the original questions. By this method, the study produced a translated Danish version of the PROMIS PF items for use in a clinical setting or for research. The standard translation methodology pointed to general types of problems in some of the original questions. The impact of such problems should be explored in quantitative studies. If a noticeable impact is confirmed, the questions should be considered for revisions. Such revisions are possible, since the item bank approach allows for new items to be calibrated and included into the bank and for old items to be retired. Translation and validation studies provide valuable data for such potential revisions.

## Abbreviations

CAT: Computer adaptive testing
DIF: Differential item functioning
FACIT: Functional assessment of chronic illness therapy
HAQ: Health assessment questionnaire
IRT: Item response theory
NIH: National Institutes of Health
PF: Physical function
PRO: Patient reported outcome
PROMIS: Patient reported outcome measurement information system
SD: Standard deviation
SF: Short form
WHO: World Health Organization
